# Brain-like illusion produced by Skye’s Oblique Grating in deep neural networks

**DOI:** 10.1371/journal.pone.0299083

**Published:** 2024-02-23

**Authors:** Hongtao Zhang, Shinichi Yoshida, Zhen Li

**Affiliations:** 1 Graduate School of Engineering, Kochi University of Technology, Kami, Kochi, Japan; 2 School of Information, Kochi University of Technology, Kami, Kochi, Japan; 3 Guangdong Laboratory of Machine Perception and Intelligent Computing, Shenzhen MSU-BIT University, Shenzhen, China; 4 Department of Engineering, Shenzhen MSU-BIT University, Shenzhen, China; University of Messina, ITALY

## Abstract

The analogy between the brain and deep neural networks (DNNs) has sparked interest in neuroscience. Although DNNs have limitations, they remain valuable for modeling specific brain characteristics. This study used Skye’s Oblique Grating illusion to assess DNNs’ relevance to brain neural networks. We collected data on human perceptual responses to a series of visual illusions. This data was then used to assess how DNN responses to these illusions paralleled or differed from human behavior. We performed two analyses:(1) We trained DNNs to perform horizontal vs. non-horizontal classification on images with bars tilted different degrees (non-illusory images) and tested them on images with horizontal bars with different illusory strengths measured by human behavior (illusory images), finding that DNNs showed human-like illusions; (2) We performed representational similarity analysis to assess whether illusory representation existed in different layers within DNNs, finding that DNNs showed illusion-like responses to illusory images. The representational similarity between real tilted images and illusory images was calculated, which showed the highest values in the early layers and decreased layer-by-layer. Our findings suggest that DNNs could serve as potential models for explaining the mechanism of visual illusions in human brain, particularly those that may originate in early visual areas like the primary visual cortex (V1). While promising, further research is necessary to understand the nuanced differences between DNNs and human visual pathways.

## Introduction

The human visual cortex is a complex system that enables visual perception through computation in cortical areas with different functions [1, 2]. Visual illusions, which are commonly used in psychology or, psychological research, have been recently used to explore explanations of visual mechanisms since they allow cross-learning with fields such as neuroscience [3–6]. Research on visual illusions provides a unique perspective on the cognitive functions and mechanisms of the visual system, as it can reveal the underlying processes that lead to the misperception of visual stimuli. A typical interpretation of visual illusions is based on a series of neural inhibitory responses that develop via the ventral visual pathway [7]. Researchers have used deep neural networks (DNNs) to model the ventral visual pathway, and mapping relationships have been built [8, 9]. Given the success of DNNs in modeling the ventral visual pathway, these networks have the potential to serve as models for the brain, allowing researchers to explore the neural mechanisms underlying visual illusions [10–12]. Despite advancements in understanding the cognitive functions and mechanisms of the visual system using DNNs and visual illusions, there is still a need for more empirical research to explore the generalizability and authenticity of DNNs as models of the human visual system across a wider range of visual illusions. The complexity and diversity of visual illusions, along with the complexity of their underlying mechanisms, necessitate the investigation of multiple visual illusions in DNNs to determine the presence of any similarities or patterns in their responses. While the phenomenon of visual illusions has been extensively studied in psychophysics [13–16], recent work has begun to investigate how these illusions manifest in deep neural networks (DNNs). These studies in psychophysics have laid the foundation for understanding the mechanisms of visual illusions, such as the role of neural inhibitory responses and the ventral visual pathway. They provide critical insights into how illusions can serve as tools to probe the workings of the visual system.

The rationale for selecting DNNs as the computational model for our study is multi-faceted and supported by existing literature. Firstly, DNNs have been shown to better predict neural activation in the primate visual cortex than other computational models [17]. Secondly, these networks have demonstrated significant potential in simulating human psychophysical tasks and have even exhibited performance metrics close to that of humans [18, 19]. Despite certain limitations, such as their inability to fully replicate some aspects of the human visual system [20, 21], DNNs have been instrumental in advancing our understanding of the human brain. In particular, some studies have revealed that DNNs can successfully mimic specific visual illusions, suggesting a close relationship between DNNs and the human visual system [5, 6]. Besides, beyond direct emulation of the visual system, the behavior of DNNs in processing visual illusions also parallels their response to adversarial examples. Adversarial examples are deliberately designed inputs meant to deceive neural networks into making incorrect judgments, similar to how visual illusions naturally mislead perception in certain conditions [22, 23]. They are all reveal potential limitations in neural network’s processing of visual information. For example, visual illusions might expose the network’s tendency to overinterpret or misconstrue visual stimuli under specific conditions, akin to how adversarial examples highlight a network’s susceptibility to certain misleading patterns. Given these aspects and the discrepancies noted in the literature, our study aims to provide a more comprehensive understanding of the underlying mechanisms and relationships between DNNs and human perception.

Most previous studies have used static geometric visual illusions in black and white to gain insight into the nature of perception [21, 24]. These graphics have limited variables and controllable factors, lacking complexity, which might not adequately reveal individual differences or delve into more complex visual processing mechanisms. In our study, we chose to use Skye’s Oblique Grating illusion [25] because it provides a unique opportunity to investigate the effects of multiple factors on the perception of visual illusions. This particular illusion allows us to systematically modulate illusion strength by manipulating the variables involved, such as color, diamond size, and positional setting. The complexity and adjustability of this type of illusion suggest that it can more comprehensively explore the brain’s processing of complex visual information, particularly in terms of the interplay between color and shape. Therefore, we can measure the DNN responses to stimuli with different illusion strengths and further explore potential mechanisms. However, whether DNNs can consistently generate realistic visual illusions remains undetermined, as opposing results have been reported for the same types of visual illusions [26]. To this end, our study aims to provide a comprehensive understanding by examining both the behavioral and neural aspects, thus bridging the gap in existing research.

Building on this foundation, our research will concentrate on addressing the limitations in the diversity of visual illusion data in extant literature. Unlike previous research that often focuses solely on whether DNNs can mimic human-like illusory behavior, our study investigates deeper into the underlying mechanisms of visual illusions within DNNs. Our study contributes significantly by employing multiple DNN models and multiple-factor visual illusions to achieve a broader understanding of visual perception. This comprehensive approach allows us to provide further empirical evidence for the applicability of using visual illusions in DNNs to understand perceptual processes in the human brain.

In an effort to tackle the problem of limited consideration of model variability in previous research, we employed multiple DNN models that showed brain-like performance according to the Brain Score [9, 27] to compare the behavior of DNNs and humans to Skye’s Oblique Grating illusion. Two experiments were conducted. The first was a behavioral task in which participants were recruited to judge the illusory stimuli. This allowed us to investigate human perception in response to Skye’s Oblique Grating illusion under different conditions, which is crucial for understanding the potential mechanisms underlying these illusions. The second experiment was performed using DNNs trained using participants’ perceptual data (images without illusion) as a dataset and tested on the illusory dataset as humans. We selected traditional networks such as DenseNet201 [28] and ResNet [29], as well as high-scoring brain-like models from the PnasNet_5_Large [30] and EfficientNet [31] series. By testing these multiple DNN models, we aimed to determine the universality of their responses to illusory images and uncover the underlying reasons for any discrepancies observed. After a series of training and testing steps, we used representative similarity analysis to observe the sensitivity of DNNs in representing stimulus data versus perceptual data.

For a deeper understanding of the DNN’s visual illusion mechanism and to address the identified concerns, we employed Grad-CAM analysis on a most representative model to directly demonstrate the feature bias of the neural network to the visual illusion stimuli. In conjunction with the corresponding partitions of the DNN, we extracted feature vectors for each module of the network. The feature vectors were then compared using a representational dissimilarity matrix (RDM) [32]. This step-by-step comparison provided a more intuitive view of the performance of a DNN for visual illusions and allowed us to better understand the factors that contribute to the discrepancies observed in previous research on DNNs and visual illusions.

## Materials and methods

### Participants

Twenty-three healthy volunteers participated in the experiment (4 female, 19 male; mean age ± SD: 27.3 ± 3.9 years). The recruitment period for the participants started on August 25, 2022, and ended on September 7, 2022. All participants had normal or corrected-to-normal visual acuity, and none had experienced an oblique illusion. The participants were tested for color blindness and were able to identify colors correctly.

### Ethical considerations

The study was supported and approved by the human research ethics committee of Kochi University of Technology and followed the relevant guidelines and regulations. All the participants provided written informed consent.

### Experimental setting

Stimuli were presented in a dark room on a Pixio PX248 Prime monitor(1920 × 1080 pixels and a refresh rate of 120 Hz), which has a physical screen size of 23.8 inches(539.94 × 400.18 × 142.56mm). Participants were kept parallel to the display by placing their heads on a calibrated chin rest (Tobii Pro AB) and maintaining a viewing distance of 66 cm from the display. The experiments were controlled using a custom Python software package, the PsychoPy toolbox [33].

### Stimuli and procedure

In this section, we detail the visual stimuli and specific steps used for the experiment. A clear experimental design is crucial, as it directly impacts the quality and interpretability of the data. The stimuli and procedures we have designed aim to accurately quantify the effects of the Skye’s Oblique Grating Illusion and provide reliable data for further analysis.

**Fig 1:**
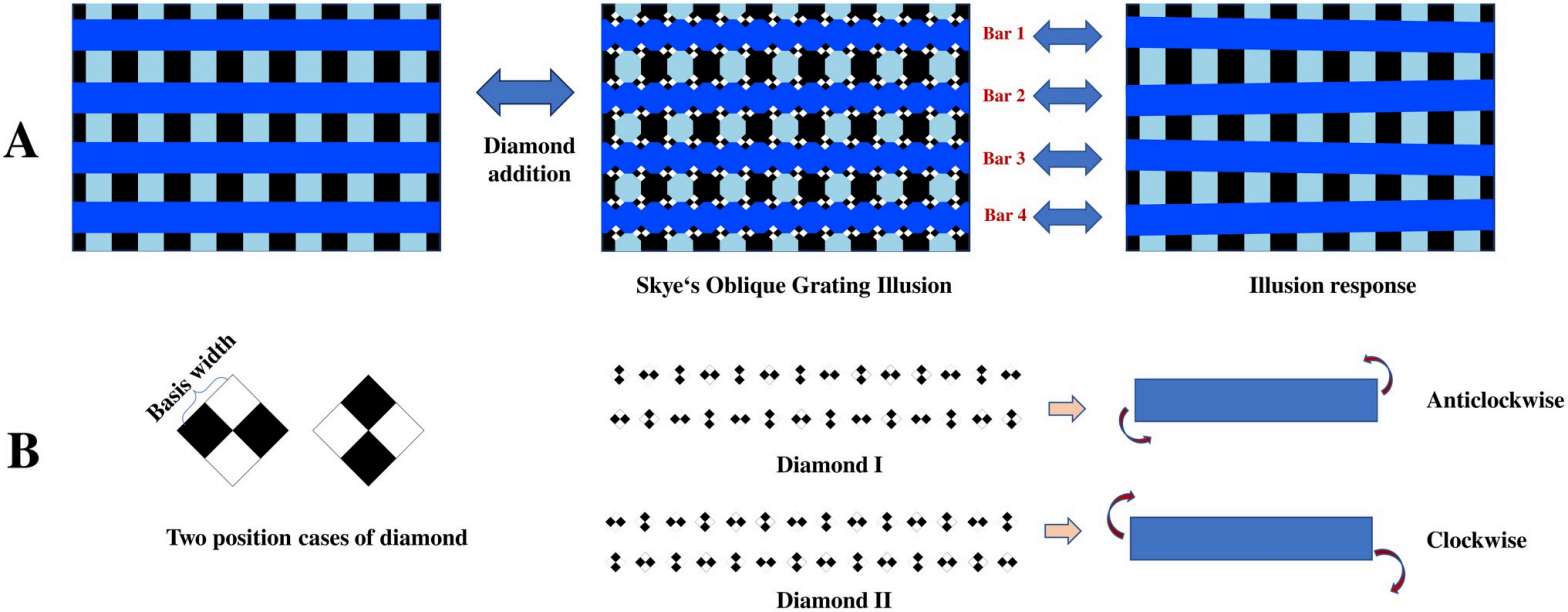
Skye’s Oblique Grating Illusion. Parallel horizontal bars create a tilted visual illusion effect when black and white diamonds are added in alternating order (A). There are two types of black-and-white diamond positional settings, with each producing opposite tilt effects (B). The tilt strength can be influenced by the width of the diamond.

To measure the strength of the illusory effect, we conducted an “adjustment” experiment. Skye’s Oblique Grating Illusion(http://www.victoriaskye.com), a variant of the visual illusion based on the Café Wall illusion, was used in our experiment [25, 34]. This geometric visual illusion, with four horizontal and parallel bars, induces the sensory illusion of tilt in the observer (Fig 1A, Skye’s Oblique Grating Illusion). The specific representation of this tilt illusion is depicted in “Illusion response” in Fig 1A. The four bars created the illusion of tilt by adding symmetrical diamond blocks consisting of two smaller black diamonds and two smaller white diamonds (Fig 1B). Diamonds can be in one of two positional settings that can trigger a clockwise or counterclockwise tilt perception (Fig 1B, Diamond I and Diamond II). The illusory tilt orientation and strength are influenced by the diamond size and positional setting.

**Fig 2:**
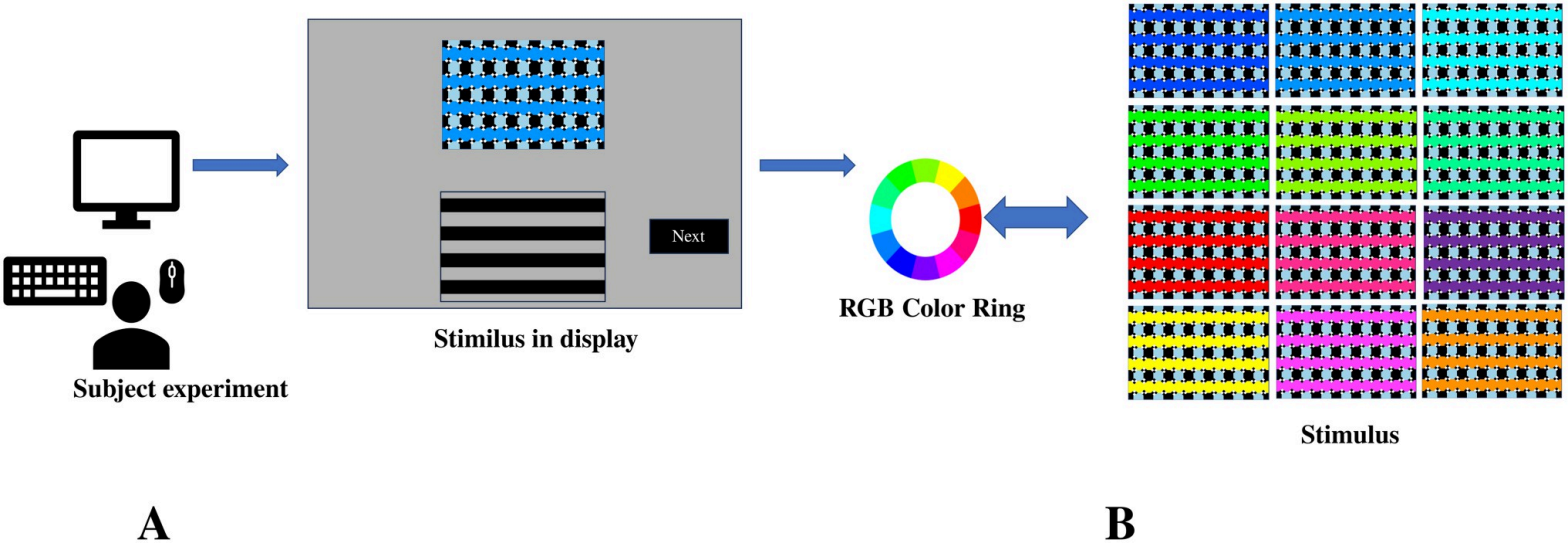
Participant experiments in this study. Participants provided feedback on visual illusions while observing stimuli on a screen (A). The stimuli were based on RGB color rings with 12 color types (B). There were six diamond width variations (5–10 pixels) and two cases of sequentially alternating diamonds, creating a total of 144 combinations of visual illusion stimuli.

In our experiment, the stimulus consisted of four long horizontal bars, each of a specific color, and diamonds with interleaved black and white colors. The background consists of interleaved black and blue vertical bars. The length of the horizontal bars was set at 560 pixels. The widths of the sides of the black and white diamonds were predetermined, ranging from 5 to 10 pixels(interval of 1 pixel). The color of the long horizontal bars was specifically selected from the 12 colors of the RGB color ring (Fig 2B, stimulus). The positions of the black and white diamonds were set in two distinct patterns(Fig 1B, Diamond I/II). In total, there were fixed 144 combinations (12 colors × 6 diamond sizes × 2 positional settings). Visually, these combinations had an approximate visual angle of 13.28 degrees horizontally and 7.61 degrees vertically.

The experiment utilized the 144 stimulus combinations, which were randomly shuffled and subsequently divided into four groups, each comprising 36 trials (with one stimulus combination per trial). A rest interval of five minutes was implemented between each group. For each trial, coresponding stimulus image was presented on the upper part of the monitor, while four black bars with adjustable orientations were presented on the bottom part of the monitor. After observing the image, participants were asked to adjust the orientation of each black bar on the bottom by pressing the keyboard, such that its orientation was perceived to be the same as that of the corresponding bar on the top (Fig 2A). Two pairs of keys were used for coarse and fine adjustments (±0.5° and ±0.1°). Simply, all participants had observed these fixed 144 illusion stimulus randomly.

To ensure participants fully understood the experimental task, detailed instructions were provided prior to the formal experiment. This was followed by 30 preliminary trials aimed at familiarizing participants with the experimental procedures. These preliminary trials were primarily focused on confirming that participants could accurately perceive the visual effects of the two directional tilts induced by the black and white diamond-shaped stimuli. After the completion of these trials, we reassured their understanding by inquiring whether they were able to observe the stimuli and adjust the angles accordingly, accurately reflecting the perceived tilt. The participants then started the experiment by pressing a key. After completing the angular adjustment of the four matching bars, the participant proceeded to the subsequent trial by clicking on the left button of the mouse. After each trial, the participants continued the experiment by pressing any key.

### Analysis

#### Illusion strength of human

We evaluated the illusion strength by analyzing the angles of four bars adjusted by the participants, referred to as “perceived angles.” These angles offer insights into how participants respond to the Skye’s Oblique Grating Illusion stimuli. To ensure the comparability of the angle data across participants, we applied absolute mean normalization. This normalization technique was chosen because it effectively standardizes the range of angle data, thereby facilitating a more accurate assessment of illusion strength. To be more specific, stimuli images composed of diamonds with two positional settings, inducing either clockwise or counterclockwise tilts, which were originally represented by positive and negative values, respectively (Fig 1B). To uniformly quantify the illusion’s intensity, we first converted the four bars’ perceptual angles to their absolute values, thereby eliminating the influence of direction and focusing solely on the magnitude of the tilt. We then calculated the average of these absolute values for the four bars, providing a measure of the overall oblique perception, which we define as the illusion strength.

#### DNNs selection

**Fig 3:**
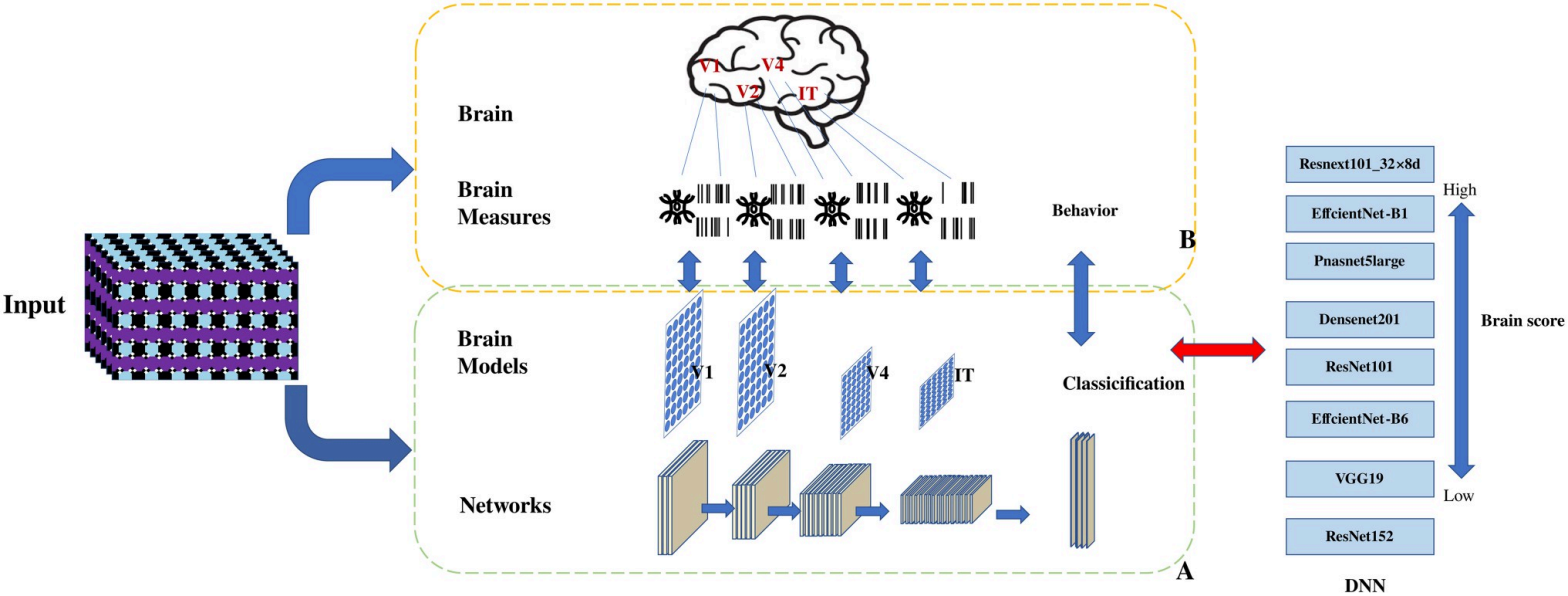
Mapping of DNNs and visual pathways in the brain. The ventral visual pathway of the brain is generally involved in object perception and recognition. The four regions from V1 to IT form perceptions (B). DNNs, as learning models for the ventral visual pathway, have multiple modules corresponding to the four brain pathway regions from V1 to IT (A). Based on the Brain Scores of the DNNs, eight models with high overall scores were selected to simulate the visual pathway. Models were ordered according to their scores, from highest to lowest.

The selection of appropriate Deep Neural Networks (DNNs) is a critical component of this study, given our aim to compare neural and computational mechanisms of visual perception. To this end, we utilized Brain Score as a guiding metric for model selection, as it offers a quantitative way to measure a DNN’s similarity to the human visual cortex, which is closely aligned with the objectives of our research. Brain Score evaluates DNNs as brain-like simulations that mimic various sublayers of the visual cortex, capturing the mappings corresponding to different regions of the visual cortex of the brain [9, 27] (Fig 3A and 3B).

It’s worth noting that the use of brain-like models in research has been a subject of ongoing debate. Some studies have found that the image recognition performance of some DNNs is negatively correlated with brain hierarchy scores [35]. However, we believe that despite this debate, the Brain Score remains a valuable metric for the scope of this study. Specifically, our focus is on understanding the underlying mechanisms of visual illusions, an area where the relevance of brain-like structures could be particularly informative.

In light of this, we selected eight DNN models based on the Brain Score, including four top-performing and complex networks (i.e., ResNeXt101_32 × 8d, EfficientNet-b1, EfficientNet-b6, and PnasNet_5_Large), which rank at the top of the Brain Score, and four more traditional and simple networks (i.e., Vgg19, ResNet101, DenseNet201, and ResNet152) (DNN, Fig 3), to compare the similarity between DNNs and human behavior. The “DNN” in Fig 3 shows the average score ranking of the models utilized. Given the uncertainty of DNNs’ responses to visual illusions, our study focuses on the performance of these models in visual illusions, allowing us to conduct a comparative analysis.

#### Binary classification

After obtaining the absolute mean normalized angular values from 144 stimuli, we categorized them into eight levels of illusion strength based on a range of 0.1 degrees. This categorization is grounded in the observed data distribution. The angular values, primarily concentrated within the 0 to 0.8 degrees range, allowed for a detailed and nuanced classification of illusion intensities at every 0.1-degree interval. Then they were divided into two groups: less than 0.4 (≤ 0.4°) were considered “No-illusion” (C1), and larger than 0.4 (0.4° ≤ deg ≤ 0.8°) were considered “With-illusion” (C2). Levels 1 to 4 were designated for “No-illusion”(C1), and levels 5 to 8 for “With-illusion”(C2). The threshold of 0.4 degrees was selected based on a clear demarcation observed in the data, where there was a noticeable shift in the participants’ perception of the illusion.

**Fig 4:**
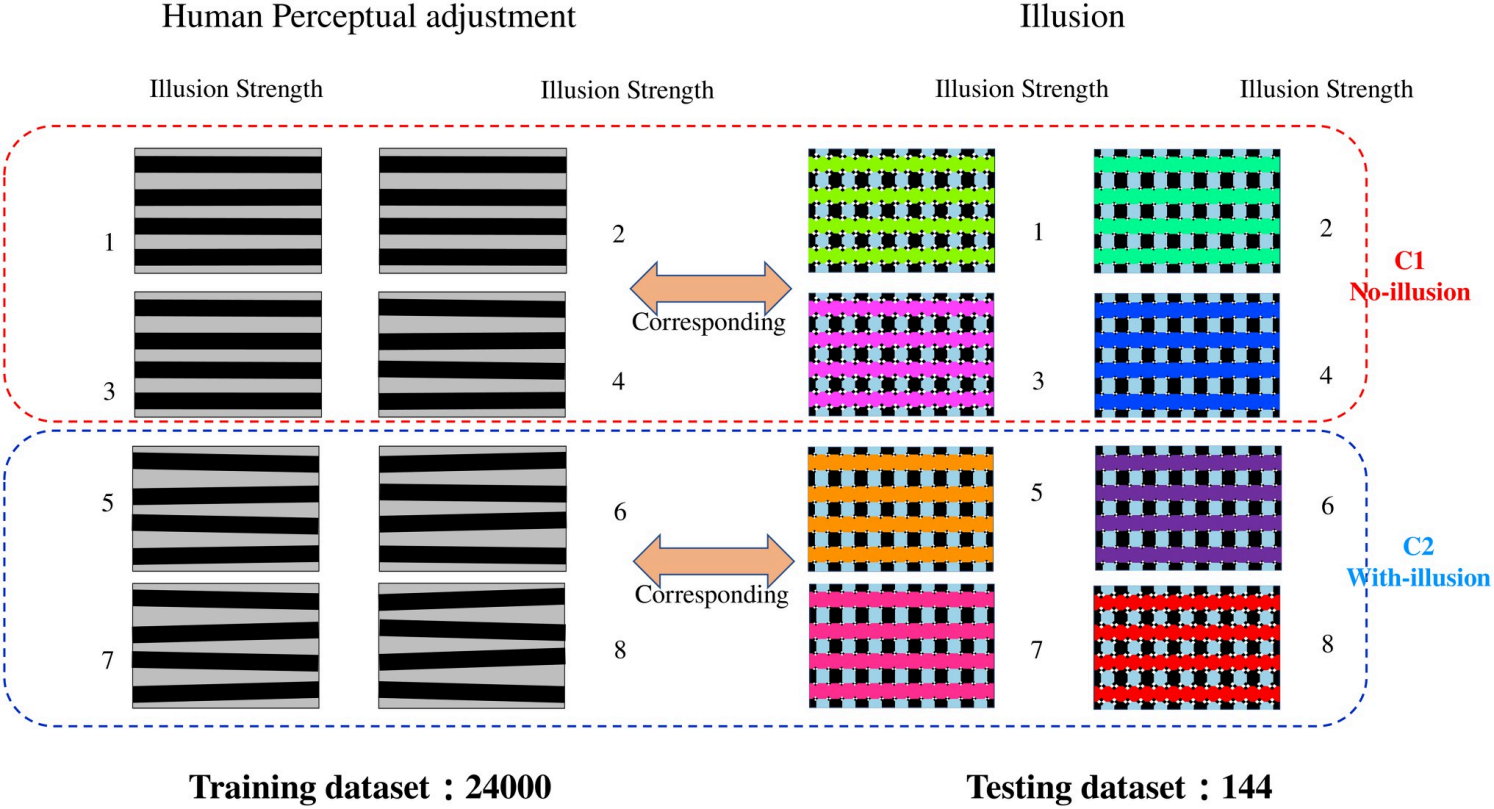
Visual illusion strength on human perceptual adjustment and illusion. The visual illusion is divided into eight ranges with a threshold of 0.1 degrees, representing eight levels of illusion intensity. On the right are the visual illusion images, corresponding to the human perception adjustment images on the left. Visual illusion images from illusion strength 1 to 4 are categorized under “No-illusion” (C1), while strength 5 to 8 fall under “With-illusion” (C2). There are a total of 144 combinations of visual illusion images, used as the test set. The human perception adjustment images on the left, featuring angled inclinations, serve as the training set for the network, comprising a total of 24,000 images.

To test whether DNNs would show “illusion,” we conducted binary classification in which DNNs were trained to perform “With-illusion”(C2) vs. “No-illusion”(C1) classification on human perception adjustment images(Fig 4 Human percetural adjustment) and tested them on illusory images used in our human behavior experiment (Fig 4 Illusion). The idea of this analysis is that if DNNs tend to predict illusory stimuli that actually contain horizontal bars to be “With-illusion”, which is similar to human behavior, it would mean that DNNs also have “illusion.”

The human perception adjustment images on the left side from C1 and C2 were used for the network training, randomly generated 3000 on 8 strength range, totaling 24,000 images with 12,000 images each for C1 and C2. The stimulus images on the right side were used as the testing set, totaling 144 images(C1:89,C2:55). All the generated images had a size of 560 ×160 pixels and were used as the training set. For the testing dataset, it consisted of 144 stimulus images that were originally presented to human participants (Fig 2B).

Simply, the DNNs were trained with actual tilted images (from the training set) and tested with images that were not tilted but had a visual illusion (from the testing set) to observe the overall test performance of the network. Five of the networks (ResNet101, ResNeXt101_32 × 8d, Vgg19, ResNet152, and DenseNet201) were obtained from torchvision, while the EfficientNet_b1 and EfficientNet_b6 were obtained from the efficientnet_pytorch package. Additionally, the PnasNet-5-Large network was obtained from the timm package.

All models underwent fine-tuning on the basis of pre-trained architectures, with the aim of adapting to and learning the characteristics of new datasets. Considering our dataset is small, our approach utilized the initial weights of the pre-trained models as a starting point, followed by comprehensive training (epoch = 100) across the entire network to better fit and learn the features of the new datasets. This process included adjusting the classification layers and implementing a learning rate decay strategy, where the initial learning rate was set at 0.001 and reduced by 10% every five epochs. To prevent over-fitting, strategies including dropout were employed, along with the Adam optimizer, weight decay, and cross-entropy loss function.

Furthermore, to ensure the efficacy of the models in skewed recognition, the training and validation sets were partitioned from 24,000 images in a 3:1 ratio. In addition, we established a separate test set of 1,200 images additionally. Post-training and validation, all models were evaluated on this independent test set, maintaining an accuracy rate exceeding 90%. This assured that the models could accurately identify categories C1 and C2 on “Human Perceptual Adjustment” before proceeding with tests on illusion images.

The entire training process was conducted on a Linux system utilizing eight Tesla V100-SXM2 (32 GB) GPUs on a DGX platform. The models were ultimately fine-tuned to perform binary classification tasks. The source code for this implementation is available at https://github.com/Vison-eden/illusion-DNN.

#### Significance test

To assess the significance of the classification results on 144 illusion images test, we performed a permutation test, a common method used in machine learning. Specifically, we trained our network using data with randomly shuffled labels and then tested the network using real label data. This procedure was repeated 1,000 times, and the accuracy levels were sorted from high to low, and finally the 95th percentile accuracy was used as the significant baseline. The baseline represents the highest performance level achieved under random conditions; therefore, any actual test performance exceeding this baseline can be considered significant, indicating that the model’s perception of visual illusions is not merely a chance occurrence.

#### Grad-CAM calculation

To further investigate the underlying feature preferences of the DNNs that lead to tilted judgments and gain insights into their responses to visual illusion stimuli, we applied the Grad-CAM technique [36] to our models. After evaluating the performance of the models on visual illusions, we selected the best-performing model and visualized its feature regions using Grad-CAM.

Grad-CAM, a deep network visualization method based on gradient localization, interprets the classification basis of the DNN model in the form of a heatmap. In our study, we compared the differences in the Grad-CAM heatmaps for images from groups C1 and C2. By analyzing the Grad-CAM heatmaps, we expect to observe a clear distinction between no illusion and illusion images in the network response. It is important to note that this comparison is qualitative, based on visual inspection, rather than quantitative analysis. This comparison will provide further evidence for the presence or absence of visual illusion effects in the DNNs and offer a deeper understanding of how these networks process illusory stimuli. The main equation is as follows:

$$\begin{array}{c} \alpha_{k}^{c}=\frac{1}{Z}\sum_{i}\sum_{j}\frac{\partial y_{c}}{\partial A_{ij}^{k}} \end{array}$$ (1)

where $\alpha_{k}^{c}$ is the weight corresponding to the *k*-th feature map, and *Z* is the normalization factor. In this equation, the indices i and j represent the row and column positions on the feature map. Next, we perform a weighted combination of the forward activation maps *A*^*k*^:

$$\begin{array}{c} L_{ij}^{c}=\text{ReLU}(\sum_{k}\alpha_{k}^{c}A_{ij}^{k}) \end{array}$$ (2)

where $L_{ij}^{c}$ is the localization map for the class *c*, and ReLU represents the rectified linear activation function that retains only the positive values.

The Grad-CAM heatmaps were then computed for images with and without tilt illusion, denoted by $L_{illusion}^{c}$ and $L_{no-illusion}^{c}$, respectively. The objective was to compare these heatmaps to investigate the relationship between the network’s response to the two types of stimulus images.

#### Representational dissimilarity matrix

Based on the model that exhibited the best performance in binary classification tests of visual illusions, we constructed a Representational Dissimilarity Matrix (RDM) [32] to compare the representational differences between real slanted images created based on human perception (Fig 4 Human Perceptual Adjustment) and illusion images (Fig 4 Illusion). In this matrix, the columns represent illusion images with illusion strength ranging from 1 to 8, while the rows correspond to the associated human-perceived slanted images.

More specifically, utilizing the model which demonstrated the best visual illusion test, we conducted feature extraction between each pair of corresponding images across intensities 1 to 8, obtaining their feature vectors. We then employed the Euclidean distance (L2 distance) as a measure of similarity between these representations to calculate their representational similarity. Each tensor has a size of 280 × 160 × 1. Furthermore, we all built the RDM across modules of varying depths. For each module, we generated an RDM with a size of 8 × 8 pixels. This unconventional approach provides a unique method for exploring and comparing the representations of different types of visual stimuli in deep learning models, thereby enhancing our understanding of the relationship between visual illusions and real perception, as well as their manifestation in neural networks. The specific formula is as follows:

$$\begin{array}{c} r_{L2}=\sqrt{\sum_{m=1}^{M}\sum_{n=1}^{N}{\left(t_{mn}^{\text{perceived}}-t_{mn}^{\text{real}\;}\right)}^{2}} \end{array}$$ (3)

$$\begin{array}{c} R_{L2}=\frac{r_{L2}}{\bar{r_{L2}}} \end{array}$$ (4)

In these equations, $t_{mn}^{\text{perceived}}$ represents the tensor of the perceived data (i.e., no-illusion images with tilted bars), and $t_{mn}^{\text{real}}$ denotes the tensor of the stimulus data (i.e., With-illusion images with horizontal bars). M and N represent the dimensions of the tensors. By normalizing the L2 distance, we can more accurately assess and compare the differences between various models and features, ultimately identifying the key features of human data that the network should be able to explain (Eq 3 *r*_*L*2_).

Our analysis focused on identifying the key features of human data that the network should be able to explain, such as capturing the specific tilt effects in the network response. By examining the RDMs at different depths of the model, we aimed to gain insights into the network’s representational characteristics and how they relate to the perception of Skye’s Oblique Grating illusion.

#### Proposed framework

Based on the analysis using Grad-CAM and the Representational Dissimilarity Matrix (RDM), we propose a comprehensive framework to integrate the exploration of visual illusions in Deep Neural Networks (DNNs). This framework aims to use the strengths of both methods to deeply understand how DNNs process respond to visual illusions, and it is expressed in following equation:

$$\begin{array}{c} S_{DNN}=F\left(G_{c}\left(I\right),R_{L2}\left(I_{perceived},I_{real}\right)\right) \end{array}$$ (5)

Where:

- *S*_*DNN*_ represents the sensitivity or response measure of DNNs to visual illusions.
- *F* represents a synthesis function that integrates the feature region visualization generated by Grad-CAM and the representational differences generated by RDM.
- *G*_*c*_(*I*) denotes the heatmap representation for class *c* obtained using Grad-CAM technique for a given image *I*, revealing how the DNN focuses on specific areas of the image for decision-making.
- *R*_*L*2_(*I*_*perceived*_, *I*_*real*_) represents the measure of representational difference between two types of images (human-perceived images and illusion images) calculated using RDM, measured by the Euclidean distance (L2 distance).

This formula shows a framework that analyzes the DNN’s response to visual illusions by quantifying the differences in internal representations and external responses when recognizing and processing illusion images. Through the analysis of *G*_*c*_(*I*), we can understand the differences in attention distribution of DNNs between “No-illusion” (C1) and “With-illusion” (C2) images; while through the calculation of *R*_*L*2_, we can quantify the perceptual differences of DNNs, especially how it distinguishes between actual physical attributes and human perception. The function *F* serves to combine these two types of analysis results, providing a more comprehensive understanding of visual illusions in DNN processing.

This integrative framework not only helps us better understand how DNNs interpret and respond to visual illusions but also reveals specific features or biases that may need to be considered in DNN design and training. In this way, we can further optimize DNN models to more closely simulate human visual processing, especially in handling complex visual phenomena such as visual illusions.

## Results

### Human behaviour results

**Fig 5:**
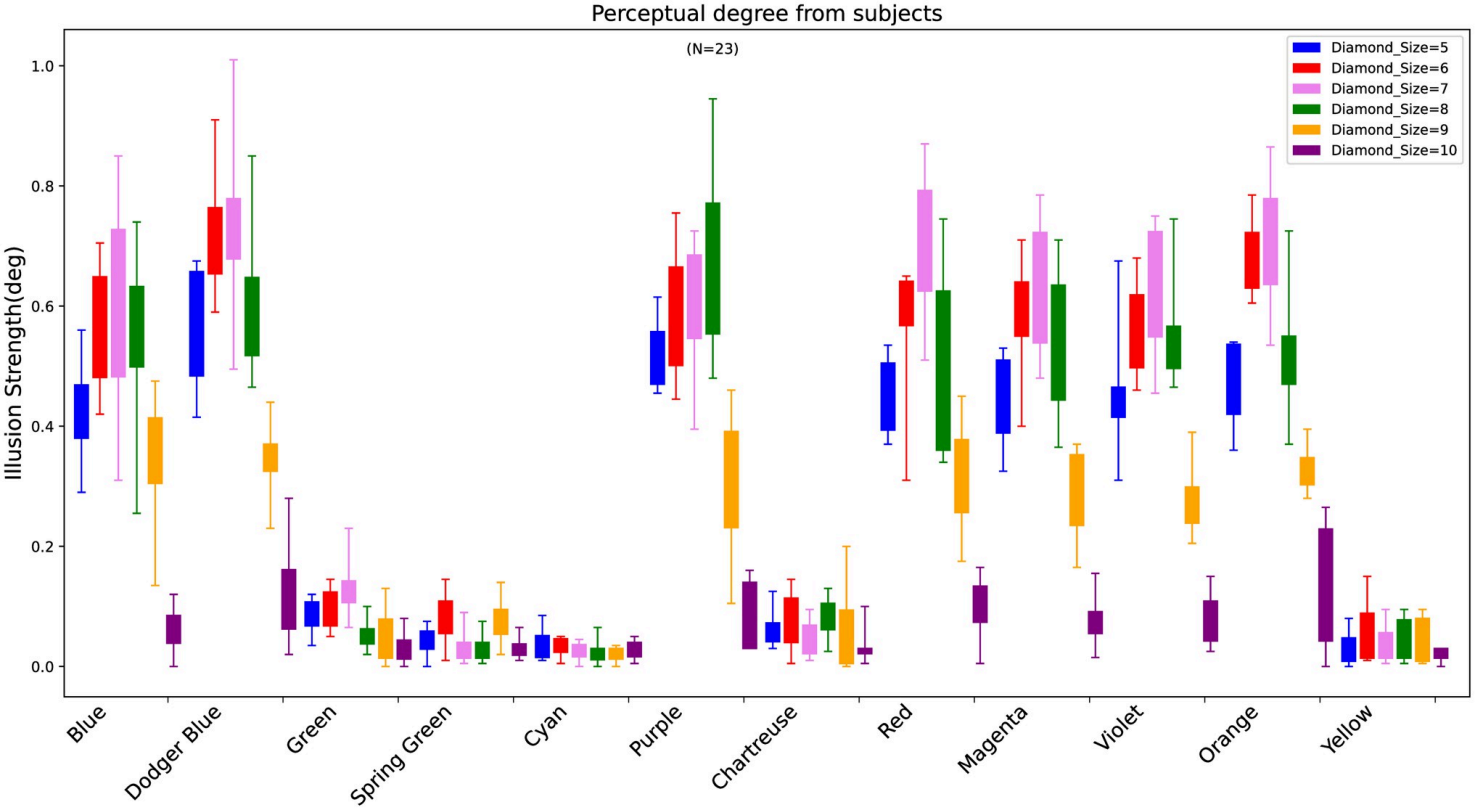
The distribution of participants’ perceived angles on 12 colors with different diamond width. The horizontal axis represents twelve colors based on the RBF color wheel, corresponding sequentially to the absolute average angles of diamond width sizes ranging from 5 to 10 pixels (with an interval of 1). Each color has a corresponding perceived angle size. The upper and lower limits in the graph correspond to the maximum and minimum angle values after adjustment, respectively.

In the previous “Methods” section, we proposed using absolute mean normalization to process the angular values of the four long bars. Fig 5 illustrates the illusion strength (average absolute values) of 12 colors under different diamond widths. The twelve colors correspond to the stimulus images shown in Fig 2B. Basically, the angular values are all within a range of 0.8 degrees divided into eight levels of illusion strength at intervals of 0.1(Level1 Level8).

Five colors (i.e., green, spring green, cyan, yellow-green, and yellow) are below 0.4 degrees across all diamond widths, while the remaining seven colors exceed 0.4 degrees at diamond widths of 5 to 8. Notably, when the diamond width of the remaining seven colors is 9 or 10, the values are less than 0.4 degrees. Using 0.4 degrees as a threshold, we categorized the results into “No-illusion” (C1) and “With-illusion” (C2), where values below 0.4 degrees belong to C1 and those above to C2. These setting were outlined in the “Methods” section.

**Fig 6:**
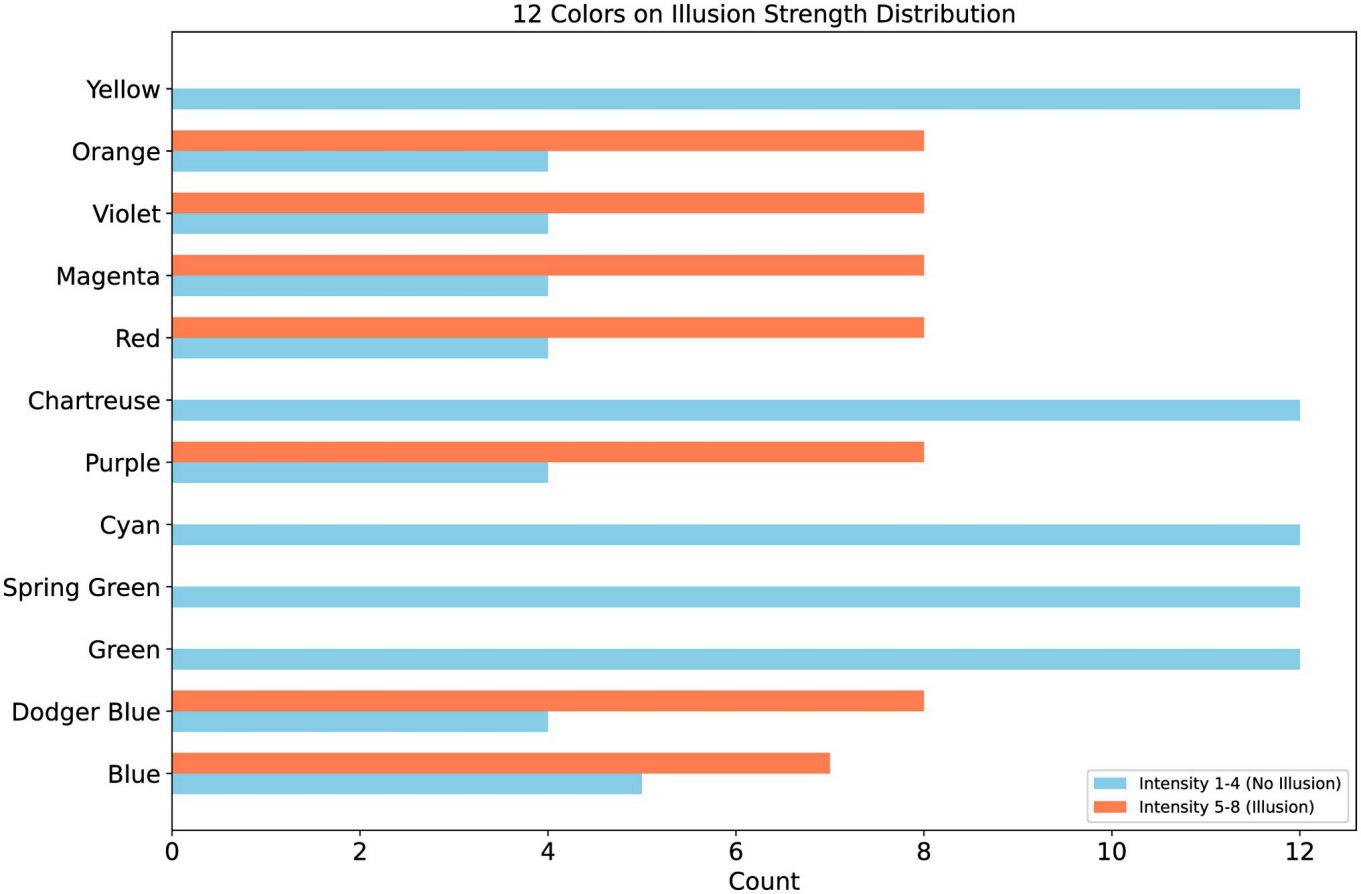
The distribution of 12 colors on C1/C2. The twelve colors correspond to their respective twelve combinations, distributed in terms of quantity within the “No-illusion” (C1) category with intensities 1 to 4, and the “With-illusion” (C2) category with intensities 5 to 8.

Furthermore, Fig 6 shows the distribution of intensities for the 12 colors under C1 and C2, providing a more intuitive representation of the specific distribution of perceived angles. Overall, out of 144 visual illusion combinations, there are 89 in C1 and 55 in C2. The five colors (i.e., green, spring green, cyan, yellow-green, and yellow) all fall under C1, while of the remaining seven colors, except for blue, the rest are distributed in C1 and C2 at a ratio of 1:2.

### Binary classification results

**Fig 7:**
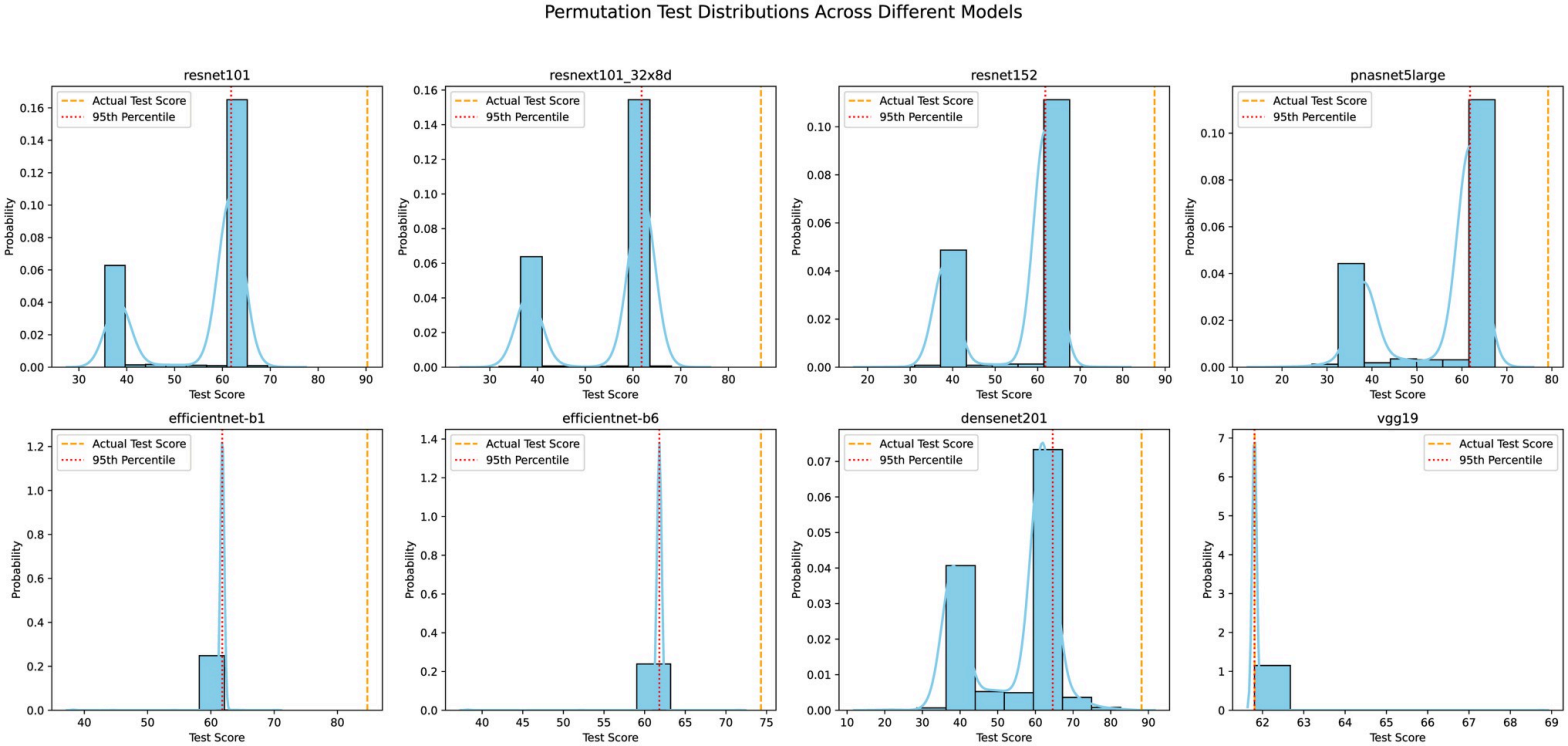
1000 times permutation test on 8 models. Examine the test accuracy distribution for eight models corresponding to real visual illusion images, as well as their accuracy after training with shuffled labels 1000 times. The horizontal axis represents the test accuracy, while the vertical axis indicates the probability distribution. The orange dashed line denotes the accuracy in testing visual illusions following correct training. Performance evaluation is based on a criterion of p = 0.05. The probability density curve reflects the potential distribution of various test performances.

Before testing eight models with 144 visual illusion stimuli images, we performed a permutation test 1,000 times. Fig 7 shows the distribution of the permutation test performance of the eight models, with each model’s distribution presented as a sky blue histogram and probability density curve in a 2x4 subplot layout. In each subplot, the actual test score of the model is indicated by an orange dashed line, while the 95% percentile of the permutation test is marked with a red dotted line, visually demonstrating the model’s performance across multiple tests. Except for Vgg19, the actual test scores of the remaining seven models were significantly higher than their 95% permutation test percentiles. This is evident in the histograms, where the actual scores’ orange dashed lines generally lie to the right of the red dotted lines (p = 0.05) with a substantial distance between them. The performance of these models far exceeds most of the randomized permutation results (label scrambling), indicating that the model’s success in testing visual illusion images is not accidental and that there indeed exists some mechanism of “visual illusion.” However, the actual test score of Vgg19 coincides with its 95% permutation test percentile, suggesting that this network may not possess the capability to correctly identify visual illusions.

**Fig 8:**
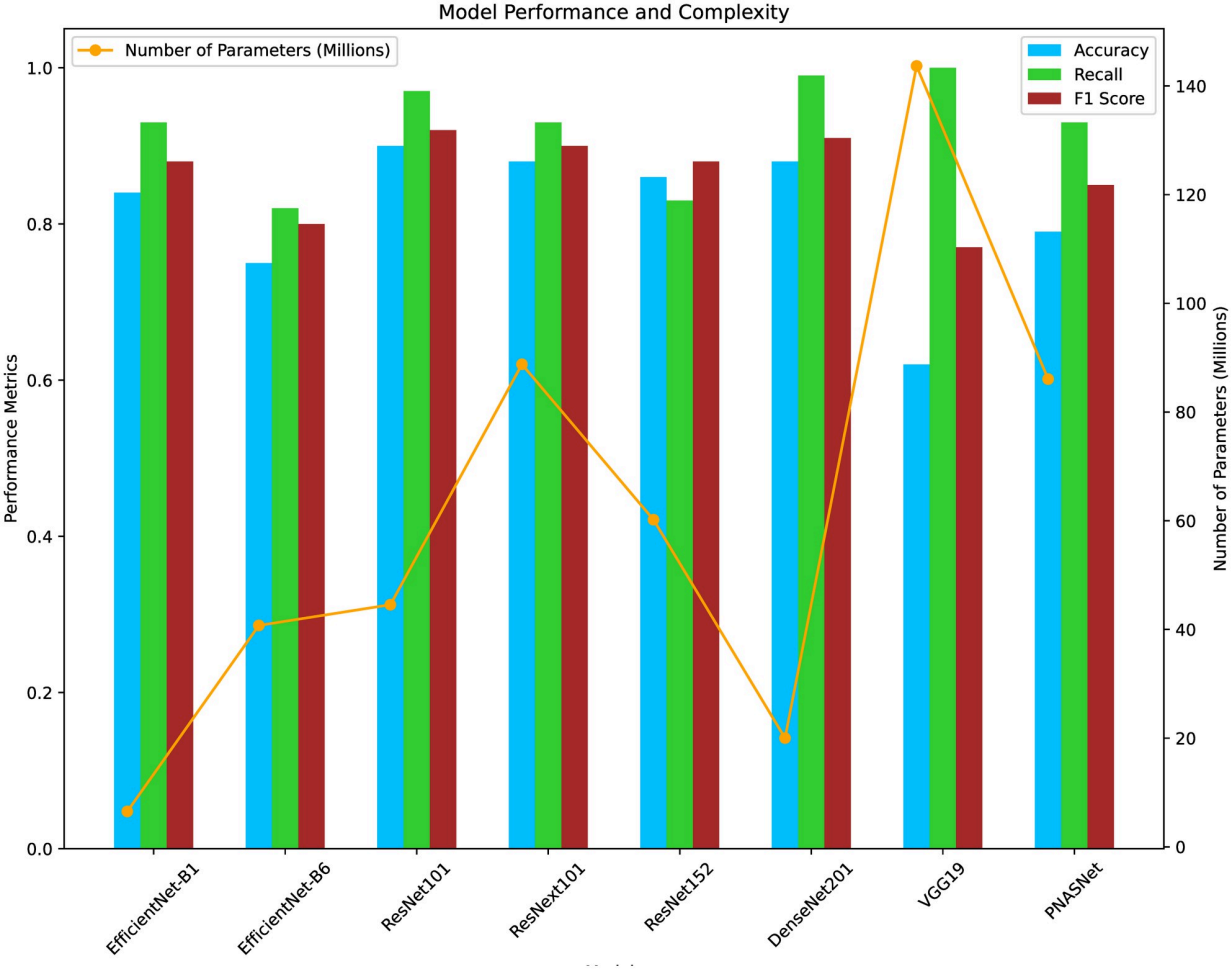
Comparative evaluation of 8 models in illusion images testing. The comparative analysis of 8 models on illusion testing using three key performance metrics: Accuracy (deep sky blue bars), Recall (lime green bars), and F1 Score (brown bars), which collectively evaluate the models’ predictive capabilities. Additionally, the chart overlays a line graph (in orange) representing each model’s complexity, measured by the number of parameters (in millions). This dual representation facilitates an understanding of how model complexity correlates with performance across different metrics.

As shown in Fig 8, the classification accuracy of the models varied considerably, and there was no clear correlation between the number of parameters and their performance in classifying visual illusions. Among all the models, ResNet101 [29] emerged as the best-performing model, achieving a classification accuracy of 90.28%. In addition to accuracy, this model also exhibited outstanding performance in recall and F1 score, further confirming its superiority in the task of visual illusion classification. This result is particularly intriguing when contrasted with the Brain Score metric we used for model selection in the “Methods” section. Despite its middle or lower-tier ranking in Brain Scores, ResNet101 exhibited the highest responsiveness to visual illusions, underscoring the complexity of neural and computational mechanisms in visual perception.

In contrast, the Vgg19 [37] network, which was considered a good model for human visual perception [38], did not show similar performance in terms of visual illusions, with a classification accuracy of only 61.81%. Other models, such as EfficientNet-b1 [31], EfficientNet-b6 [31], ResNeXt101_32 × 8d [39], ResNet152 [29], DenseNet201 [28], and PnasNet_5_Large [30], exhibited varying degrees of classification accuracy, ranging from 74.31% to 88.20%. However, in terms of recall, DenseNet201 performs slightly better with a score of 0.99 compared to ResNet101’s 0.97. Additionally, it has fewer parameters than ResNet101.

**Fig 9:**
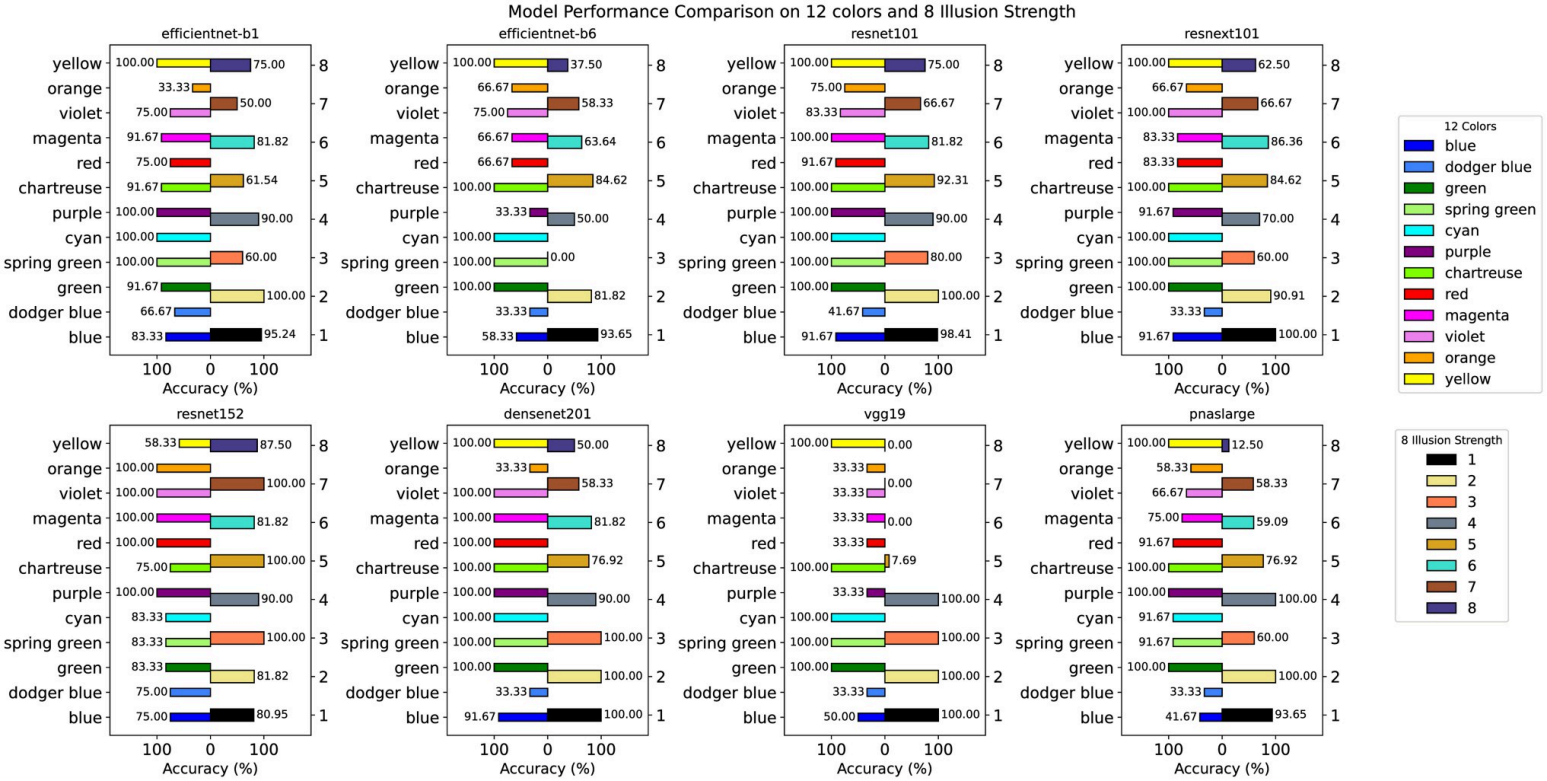
Comparative analysis of eight models: Performance across varied color and illusion intensities on Skye’s Oblique Grating. Color Categories (Left Axis): This legend explains the different color bars on the left Y-axis representing various color categories. Each color bar represents the recognition accuracy of the corresponding color in different models. Illusion Intensity Categories (Right Axis): This legend explains the different color bars on the right Y-axis representing levels of illusion intensity. Each color bar represents the recognition accuracy of the corresponding level of illusion intensity in different models.

Morever, we further demonstrated the performance of eight models under twelve colors and eight intensity levels (Fig 9). From the perspective of color recognition, most models showed significantly high accuracy in certain specific colors, especially in colors like ‘green,’ ‘spring green,’ ‘cyan,’ and ‘yellow,’ where many models achieved a 100% accuracy rate. However, performance was poorer in colors such as ‘blue,’ ‘magenta,’ and ‘purple,’ suggesting that some models have stronger adaptability to certain color ranges. Notably, EfficientNet-B1 and ResNet101 exhibited higher accuracy across most colors, reflecting their potential strength in processing natural hues, while EfficientNet-B6 and Vgg19 showed lower accuracy across various colors, particularly in ‘orange’ and ‘purple,’ indicating their insensitivity in distinguishing specific hues. Almost all models performed unsatisfactorily in ‘royal blue,’ possibly pointing to a common recognition challenge with this particular color.

In terms of intensity recognition, most models seemed more accurate in handling moderate intensity levels (such as 4 or 5), while accuracy generally decreased at extreme intensity levels (such as 1 or 8), indicating challenges these models face in processing subtle or overly obvious intensity changes. ResNet152 and DenseNet201 demonstrated higher accuracy across most intensity levels, especially from medium to high levels, while ResNet101 also performed well at moderate intensity levels (such as 3 to 6), showing its balanced capability in processing moderate visual changes. On the other hand, Vgg19 and PNASLarge performed poorly at extreme intensity levels, reflecting their insufficient sensitivity in recognizing subtle changes or overly strong visual effects, and EfficientNet-B6 also showed poor performance at low intensity levels, suggesting its limitations in processing fine visual changes.

Given the superior performance of ResNet101 in recognizing visual illusions, further investigation and analysis of this model are warranted. We examined ResNet101 in greater detail to explore its characteristics and capabilities within the context of visual illusion classification, as explained in the following sections.

### Grad-CAM visualization

To explore the slight responses of deep neural networks (DNNs) to visual illusions and assess their resemblance to human perception, we utilized Grad-CAM visualizations [36]. Our focus was on understanding how these networks react to both “With-illusion” (C2) and “No-illusion” (C1) stimuli and visualized feature preferences across eight distinct models for this purpose. This technique is an extension of our methodology, where we hypothesized that if DNNs misclassify horizontal bars in illusory stimuli as non-horizontal, akin to human behavior, it would indicate that DNNs are also susceptible to the “illusion.”

**Fig 10:**
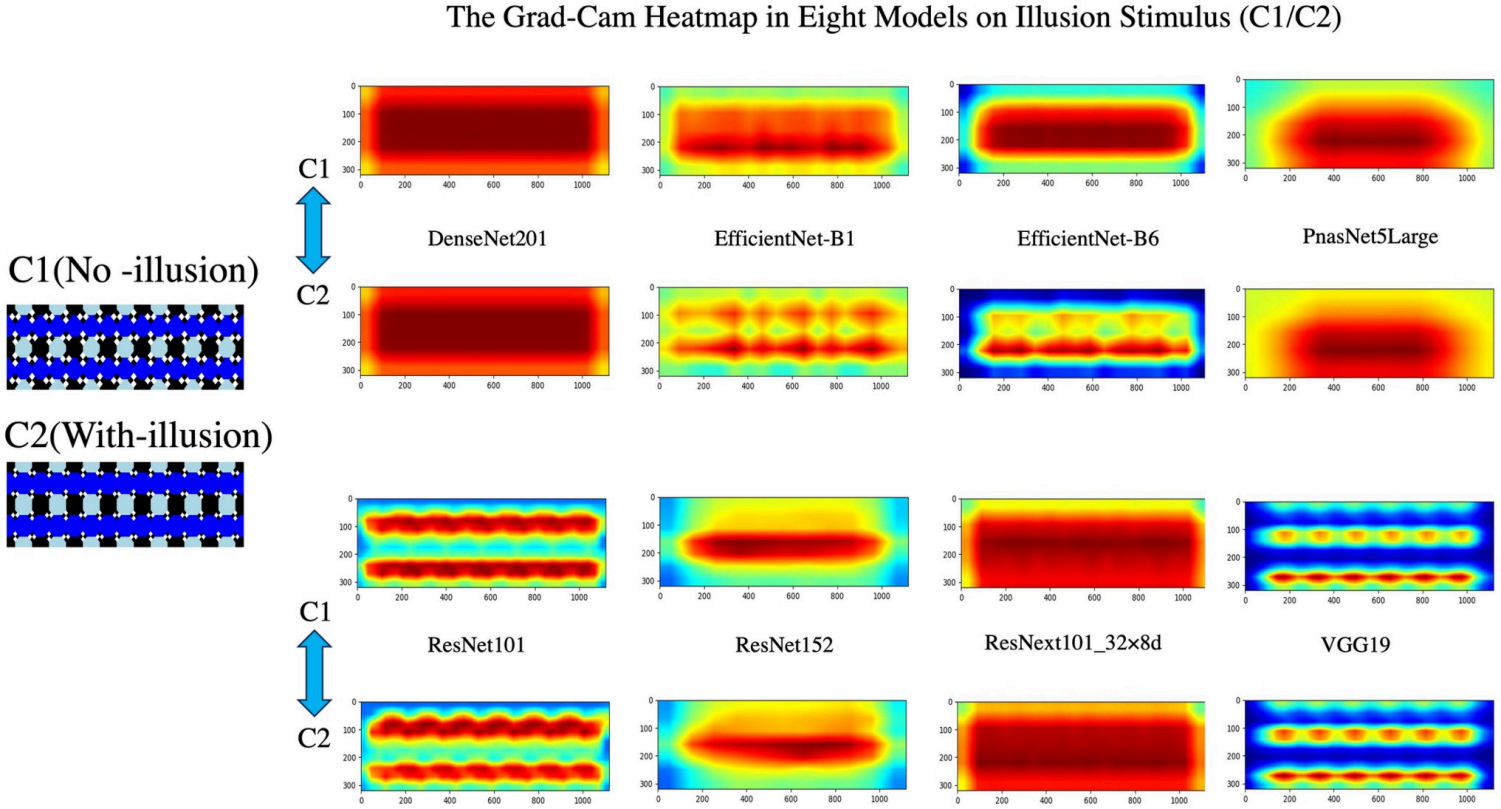
The features heatmap of C1/C2 illusion images on 8 models. Feature preferences of eight models under No-Illusion (C1) and With-Illusion (C2) conditions. The stimulus features for C1 are displayed above each model, while those for C2 are shown below.

As shown in Fig 10, the models exhibited diverse feature preferences. When exposed to “No-illusion” (C1) stimuli, most of the models gravitated towards a global feature focus. However, specific models like ResNet101 and Vgg19 deviated from this trend, displaying a preference for stripe-shaped features. Interestingly, when subjected to “With-illusion”(C2), several models demonstrated a shift in feature preferences compared to C1 stimuli. Some models exhibited more pronounced curvatures in features, while others showed a tendency for features to decompose into multiple regions. Models like Vgg19 and DenseNet201, however, remained largely invariant to the type of stimuli.

**Fig 11:**
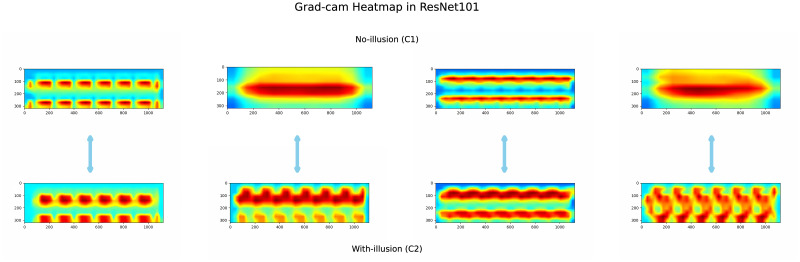
The feature heatmap of ResNet101 on Skye’s Oblique Grating Illusion. We used back-propagation of gradients to compute weights to visualize the feature bias of the DNN on two stimulus images of C1 and C2. The ResNet101 yielded totally four corresponding features on no illusion (C1) and illusion (C2).

Based on the ResNet101, to further explore the “illusion” in inner network, we employed the ResNet101 network to examine the 144 illusory images from our test dataset. This analysis revealed four distinct categories of feature preferences when these illusory images were tested. Specifically, these preferences are pairs of feature biases that emerged from examining the 144 illusory images. As illustrated in Fig 11, the feature maps for non-illusory stimuli (“No-Illusion” C1) and illusory stimuli (“With-Illusion” C2) are presented in two corresponding rows. The feature heatmap for C1 shows patterns such as block features in two rows, a large central block, or two long, curved bars. In contrast, the corresponding C2 heatmap indicates a fusion of the two-row block features from C1, or the decomposition of the large central block into a curved pattern, or longer, more curved bars. These differences suggest how ResNet101 discerns visual illusions, with the feature biases for illusory stimuli aligning closely with those for non-illusory stimuli, indicating a consistent trend in feature extraction across both types of stimuli.

### Representational dissimilarity matrix results

In this section, we delve deeper into the representational characteristics of deep neural networks in response to visual illusions, with a particular emphasis on the ResNet101 model. This exploration aligns with our methodological framework, where we employ Representational Dissimilarity Matrices (RDM) to compare network representations with human behavioral data.

In the classification of “With-illusion” (C2) and “No-illusion” (C1), we further reveal the performance differences of various deep learning models in processing visual illusion recognition tasks by calculating the L2 distance in correctly identifying C1/C2 versus incorrectly identifying C1/C2. By combining the overall accuracy of the models with the analysis of average L2 distances at various layers, we can identify the strengths and weaknesses of each model in feature extraction and classification of visual illusions.

**Fig 12:**
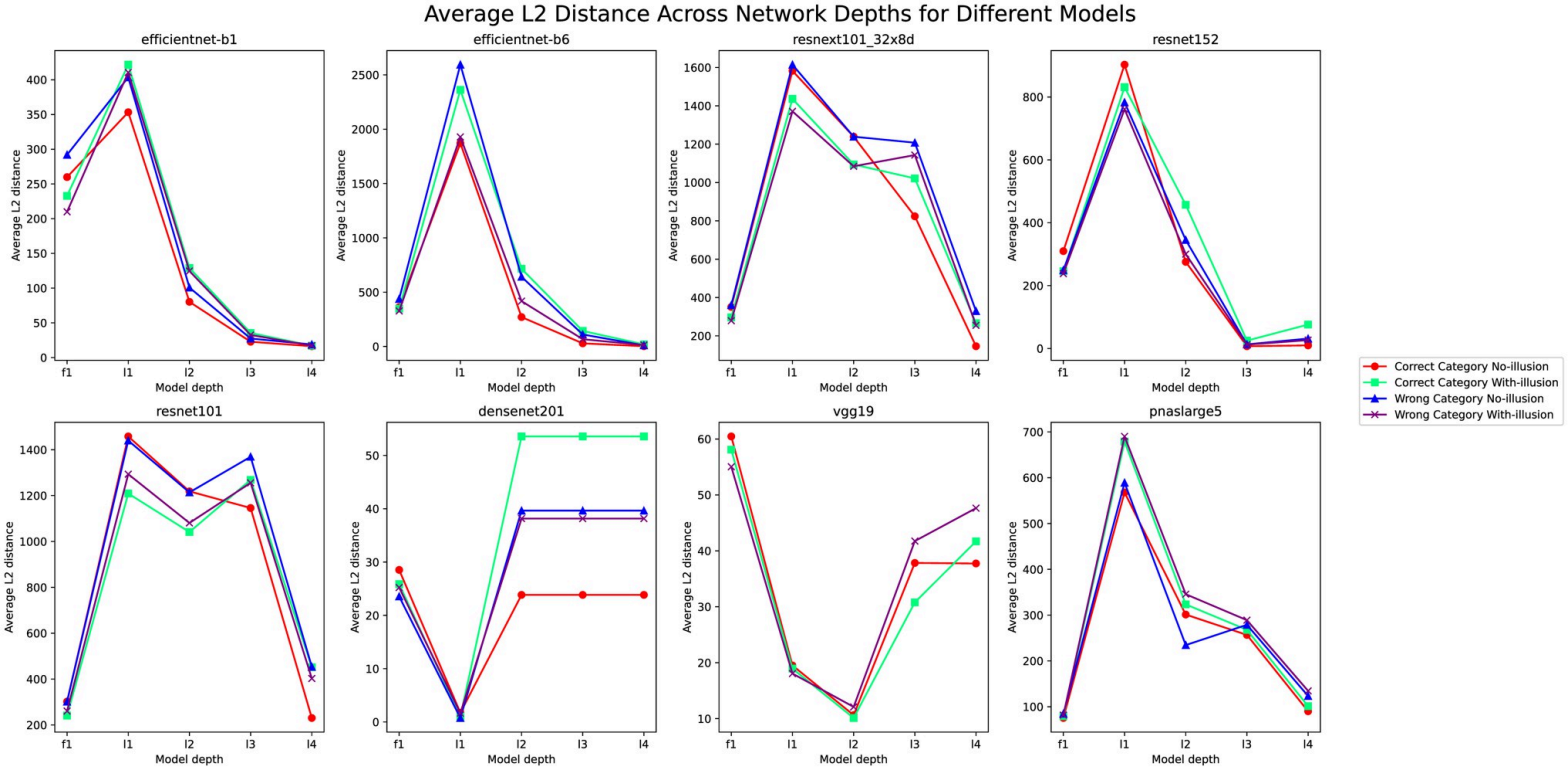
Comparative analysis of the variation in average L2 distance across network depths for different models. Variations in total representational similarity(Average L2 Distance on human perceptual adjustment and illusion) across different network depths in DNNs. The legend represents different classification conditions. Red circles indicate correctly classified data points under condition C1, while spring green squares correspond to correct classifications under condition C2. Blue triangles mark instances of incorrect classification under C1, and purple crosses represent incorrect classifications under C2. The horizontal axis denotes the respective depths of the network.

Observing from Fig 12, when combining the average L2 distance data of the models at different network depths with their overall classification accuracy, we notice significant differences in how different models process visual illusion features. EfficientNet-B1 and EfficientNet-B6 show larger L2 distance variations at shallower layers, suggesting a limited differentiation capability for visual illusions at the primary feature extraction stage, but exhibit improvements at deeper levels. Meanwhile, ResNet101 and ResNext101 demonstrate higher accuracy rates (0.90 and 0.88 respectively), indicating better overall performance in handling visual illusion images, although they show similarities in processing incorrectly classified samples at certain levels to those correctly classified. In contrast, the low accuracy of Vgg19 (0.61) and its higher L2 distances for incorrectly classified samples at various levels clearly indicate its deficiencies in recognizing visual illusion features. ResNet152, DenseNet201, and PNASNet exhibit a certain balance in terms of accuracy and L2 distance, but there is still room for improvement at certain depths.

Having established this broader perspective, we now hone in on ResNet101, which has demonstrated superior performance in recognizing visual illusions. We employed the Euclidean (L2) distance as a similarity metric for representations and generated an RDM for each module in this model. The analysis aims to elucidate the network’s representational characteristics in relation to Skye’s Oblique Grating illusion. Unlike regular RDM construction, we utilized the L2 distances between pairs of corresponding visual illusion images and human-adjusted images to construct the RDM (Fig 4).

**Fig 13:**
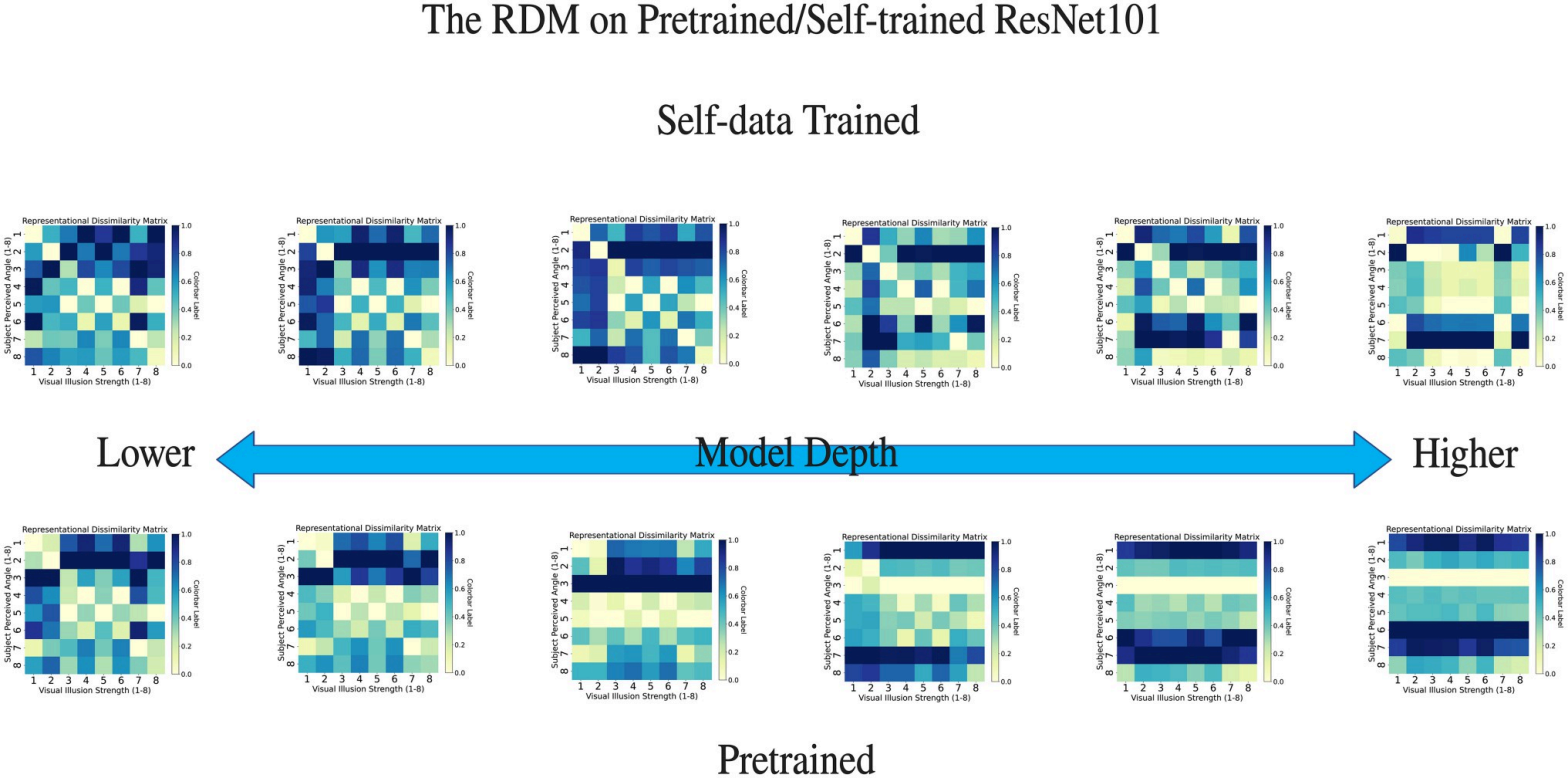
Representative dissimilarity matrix (RDM) on different model depths of ResNet101. We utilized the top-performing illusion response, ResNet-101, in the RDM for testing. Each RDM corresponds to network depth. The horizontal coordinate represents the stimulus images by illusion strengths 1–8, and the vertical coordinate represents the perceived angle images by illusion strengths 1–8. Network depth increased from left to right. The RDM group for Self-data Trained corresponds to the scenario where training is conducted with tilted images, while the RDM group for Pretrained corresponds to the RDM under the condition of pre-training loading (without training).

Fig 13 displays the RDMs of ResNet101 at various network depths, sized 8x8. Each row of an RDM represents an image adjusted according to human perception, and the columns represent visual illusion images, with numbers 1 to 8 corresponding to the pre-set intensities from 1 to 8. This arrangement allows us to visually observe their similarities and differences, aiding in understanding the information representation process within the neural network. The metric in the RDM matrix is based on the L2 distance, represented in the figure by a color scale ranging from 0 to 1 to indicate the actual L2 distances. The intensity of the color denotes representational similarity, with darker colors indicating lower similarity and lighter colors indicating higher similarity.

Considering the pairwise corresponding visual illusion and human perception data images, we focus on the similarities along the diagonal of the RDM. In the experimentally trained ResNet101 (Fig 13 Self-data Trained), we observe high diagonal similarities at shallower network depths. This high level of representational similarity indicates that human perception images of illusion intensities 1-8 and visual illusion stimuli images are closely correlated in the ResNet101 model. The neural network demonstrates a response to visual illusion stimuli images akin to human perception, effectively capturing the underlying features associated with these different illusion intensities.

As the network depth increases, we notice a darkening of the color around the diagonal area in the RDM, indicating a decrease in non-corresponding representational similarity. This finding is crucial for our research as it provides evidence supporting our earlier hypothesis in the “Methods” section, suggesting that DNNs might exhibit sensitivity patterns to illusions similar to those in humans. A significant difference is observed in the fully connected layers, characterized by darker colors.

**Fig 14:**
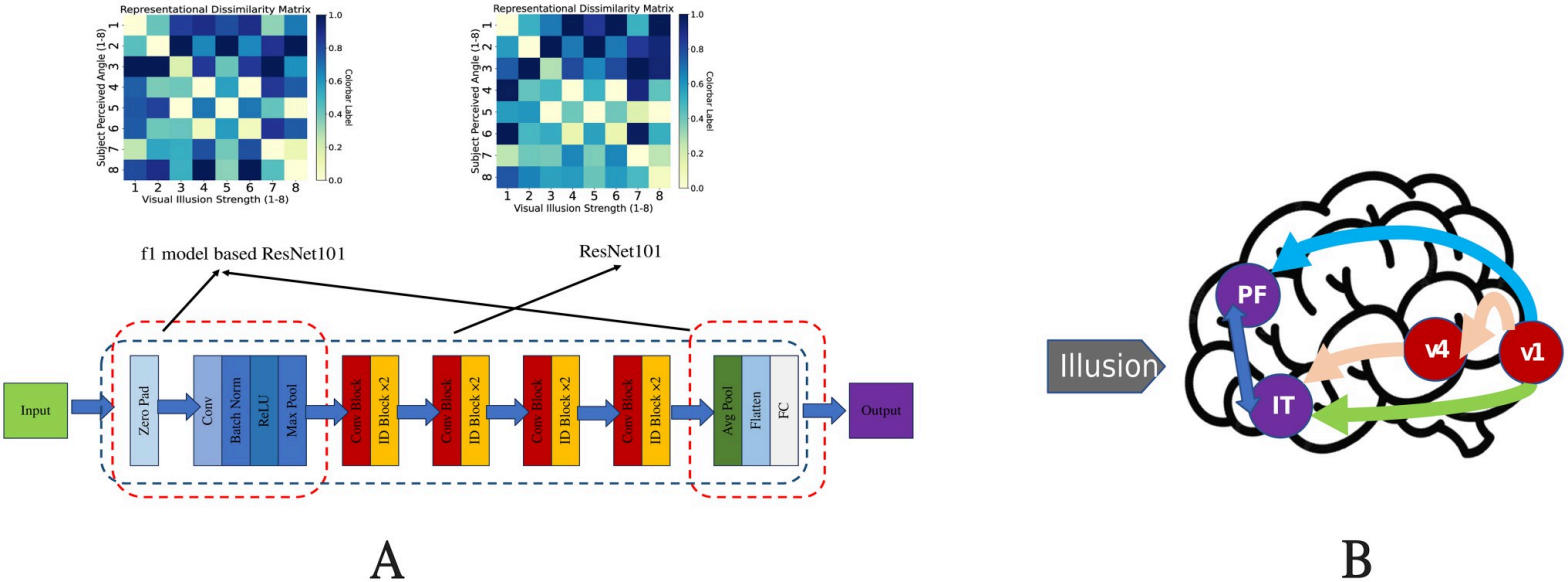
Representational dissimilarity matrix (RDM) based on ventral access partitioning. (A) A comparison of the RDMs for a single network architecture and complete network architecture. (B) The potential vision of the visual pathway. The red dashed region in panel A represents the F1 module network built by the ResNet101 shallow module and trained by the same dataset. The dashed blue area represents the entire network of ResNet101. Both represent the V1 and ventral pathways (V1 to IT) in panel B, respectively.

Additionally, a similar trend is observed in the pretrained ResNet101 (untrained); however, the reduction in representational similarity occurs more rapidly. Interestingly, when shallow layers are trained separately as different network architectures and then tested, they exhibit RDMs similar to those trained with the full architecture (Fig 14A), indicating that responses to visual illusions are highly pronounced in the shallow layers of ResNet101.

## Discussion

In the present study, we aimed to shed light on the relationship between human visual illusions and neural network performance. The underlying mechanisms of Skye’s Oblique Grating illusion in both human perception and Deep Neural Networks (DNNs) were explored. Our findings support the idea that DNNs can mimic certain aspects of human visual perception, although differences remain.

We assessed the relevance of DNNs in understanding cognitive brain tasks by comparing the performance of various DNN models with human perceptual data using similar visual-illusion stimuli. The brain score model emerged as a crucial element in simulating brain-like performances [27].

Our experiments found that networks with high similarities to the brain in our illusory task do not have to be complex. This finding is consistent with some previous studies. Nonaka et al. [35] found that some simple networks are closer to the human visual cortex, which sheds light on the intriguing fact that there may be some trade-off between network complexity and brain similarity in neuroscience research.

However, it is important to emphasize that in our study, the ResNet101 model, which has a residual module and skip-and-branch connections, exhibited the best visual illusion response among the tested networks (Fig 8). In contrast, Vgg19 showed the poorest performance, while the other advanced models (i.e., ResNet152, ResNeXt101_32 × 8d, PnasNet_5_Large, EfficientNet-b1, and EfficientNet-b6) achieved varying levels of performance; however, none surpassed ResNet101.

Moreover, expect for Vgg19, all models exhibited a distribution at p = 0.05 in the permutation tests for test accuracy (Fig 7), indicating that the models’ responses in the identification tests were not due to chance, but rather represented an “illusion”. Vgg19, however, failed to recognize or understand illusion images.

When considered in conjunction with Fig 12, EfficientNet-B1 and B6 displayed significant fluctuations in L2 distance at shallow layers, suggesting a weaker ability to differentiate illusions at the initial feature extraction stage. However, this ability appears to improve with increased network depth, indicating that these models process illusions more effectively during more complex feature integration stages. In contrast, ResNet101 and ResNext101 demonstrated higher overall accuracy, possibly due to their unique network architectures, such as residual connections, which facilitate deeper information transmission and complex pattern analysis. On the other hand, Vgg19’s performance in processing illusions was relatively poor, likely due to its simpler network architecture. ResNet152, DenseNet201, and PNASNet exhibited a balance between accuracy and L2 distance, although there is still room for improvement at specific layers.

Similar to feature extraction, we used the feature vector of the image in the DNN to determine the visualization of its visual illusion performance. DenseNet201 and ResNext101_32x8d, which performed relatively well in tests, along with PnasNetSLarge, displayed feature preferences for entire regions in C1 and C2(Fig 10). This reflects the models’ inability to precisely understand the slanted changes in illusions. Conversely, although EfficientNet-B1 and EfficientNet-B6 also showed similar feature preferences in C1, they exhibited multiple small regional features in C2. ResNet101 displayed curved longitudinal features, with the difference between C1 and C2 being that the features in C2 were more curved. Vgg19 also had a similar longitudinal feature preference, but with no significant change between C1 and C2. According to its performance of permutation test and lowest accuracy on testing, it indicated that simple networks like Vgg19 are not more brain-like. Basically, the difference in feature preferences between C1 and C2 seems to be an expression of the network generating illusions.

As demonstrated in Fig 11, we then deeply analyzed the feature tendencies of stimulus images in ResNet101 for both C1 (No-illusion) and C2 (With illusion). The images were presented in pairs, corresponding to each other. In the C1 (no illusion), the stimulus images displayed regular but disconnected block-like features in the DNN, appearing as elongated blocks. In contrast, the C2 (illusion) stimulus images showed connected block features, exhibiting a curved tendency. Our findings indicate that when the DNN exhibits a performance similar to human perception in visual illusion judgments, it demonstrates a preference for wave trend. Alternatively, instead of focusing on holistic features, the approach involves dividing the feature into multiple interconnected blocks, which also exhibit a preference for curved feature representations. This bending variation in the feature representation leads to the emergence of visual illusion responses in the DNN.

As shown in Fig 13, DNNs show a high bias response in the early layers; however, this performance diminishes as the network deepens. Engilberge et al. [40] states that DNNs are more color-sensitive in earlier modules, and as the network deepens, the DNN becomes more sensitive to the category rather than color. Interestingly, our visual illusion stimuli, which differed in color, showed varying illusions of tilt bias or none, influenced by colors.

Further comparison of color accuracy distributions (Figs 5 and 9) shows that subjects maintained high illusion performance for seven colors (blue, Dodger Blue, purple, red, Magenta, Violet, orange). In DNN illusion tests, the models were not “sensitive” to blue and Dodger Blue, showing relatively low accuracy, while the remaining three colors (red, Magenta, Violet) performed well in all seven models. Orange maintained high accuracy in Resnet101 and Resnet152, but DenseNet201, despite its overall good performance, showed very low capability. In general, the models with higher test accuracy demonstrated a balanced sensitivity to colors, which is related to the complexity of illusions. For instance, ResNet101 and ResNet152 exhibited high accuracy in processing orange, reflecting these models’ strong recognition capabilities for specific colors. Meanwhile, despite DenseNet201’s overall good performance, it unexpectedly underperformed in recognizing orange, suggesting that even high-performance models might have limitations in processing certain colors. These differences among models, likely due to their varying architectures, indicate the need for more attention to different color features during model training and optimization. Moreover, these findings also imply that illusion images hold potential value for testing and improving the visual processing capabilities of deep learning models.

In addition, Fig 14A shows that when we trained the shallow depth in ResNet101 as an individual network, its RDM was high, similar to ResNet101’s shallow depth. This indicates the origin of “visual illusion,” probably produced in early layers in DNNs, which may correspond to the human primary visual cortex (V1). This result is consistent with those of previous studies. Several studies have shown that neural responses in the V1 are modulated by spatial and temporal context and related to the perception of visual illusions, such as the tilt illusion and tilt aftereffect. Studies have demonstrated a significant relationship between the magnitude of neural suppression in the V1 and the strength of these illusions [41, 42] (Fig 14B). Contrary to the conventional understanding that DNNs model the ventral stream of the visual system, our findings point to limitations in this analogy. Specifically, higher layers of the DNNs, generally assumed to correspond to the Inferior Temporal (IT) layer in humans, did not exhibit illusion-like judgments, unlike human perception [8, 43].

Basically, our research on the oblique grating illusion based on cafe wall variants, by quantifying visual illusions, enables Deep Neural Networks (DNNs) to learn and judge visual illusions, discovering that DNNs have unique visual illusion response patterns. Similarly, the studies by Ward et al. (2019) [21] also categorized and quantified visual illusions using human perception data, then tested the responses of DNNs with these quantified standards to explore the sensitivity and reaction of neural networks to visual illusions. As for the method, our research is similar to previous studies in the objective of understanding the visual illusion responses of DNNs. We both utilized the quantitative methods of human visual illusions, applying them to neural networks to evaluate their response capabilities to different visual illusions. Moreover, we quantified whether DNNs experience visual illusions by extracting the feature vectors of DNNs for illusion images and calculating the Euclidean or cosine distance between the feature vectors of perceived images and illusion images. Especially, at present, visual illusions like the Müller-Lyer illusion, or the Hermann grid illusion and color assimilation illusion, all have shown human-like visual illusion performances in some DNN models [3, 5, 21].

In the studies by Ward et al. (2019) and Sun, Eric D. (2021) [5, 21], it was found that the pre-trained Vgg19 displayed responses to visual illusions, such as length changes (Müller-Lyer illusion) and the perception of flickering dots (Hermann grid). Most of these studies were based on exploring and testing with pre-trained models. The issue with this approach is that, although pre-trained models with good classification performance can exhibit high visual task performances, models specialized for specific tasks do not necessarily show uniform visual illusion responses. This was also emphasized in the research by Ward et al. (2019) [21], indicating that models seem unable to understand other geometric visual illusions, showing very low response. In other words, in our research, features of visual illusion misjudgment, such as the angle of bars, are difficult for neural networks to understand what is oblique, with the model focusing more on overall features to express their visual understanding. However, in this study, although Vgg19 was trained to understand oblique bars, it still did not show the performance of visual illusion. This further implies the potential limitations of the model in image processing. Moreover, Gomez-Villa et al. (2019) [3] trained neural networks to learn color-related visual tasks, finding that models exhibited color visual illusions (such as color assimilation). Similarly, we also trained based on specific visual illusion tasks, and DNNs showed significant visual illusion performances.

In Fig 13, we explored the similarities and differences under pre-trained and self-trained data. The early layers are essentially consistent, with significant differences starting from the middle layers, although both showed irregular representational similarity in the last layers, i.e., no visual illusion response. The response to visual illusions varies across different models and training backgrounds, and although our research explains through RSA and CAM visualization, the mechanism of visual illusions still needs to be considered for the limitations of the training dataset of model. Our findings emphasize the need for a comprehensive consideration of the differences in performance between pre-trained and self-trained models in visual illusion research.

Although this study offers preliminary insights into the capabilities of neural networks in processing visual illusions, our approach has certain limitations. Notably, our experiments primarily focused on specific model instances without encompassing multiple instances of the same architecture. Specially some study indicated that neural networks can exhibit significant differences in internal representations across instances due to the randomness of initial weight assignments during training [44]. Consequently, our findings may primarily reflect the characteristics of the specific models tested, and may not necessarily apply to all instances.

Future research should include testing multiple model instances under the same architecture to verify the generalizability and reproducibility of our findings across different instances. This will contribute to a deeper understanding of the capabilities of neural networks in recognizing visual illusions and provide guidance for designing more accurate visual processing models.

In conclusion, this study has shed light on the relationship between human visual illusions and neural network performance. While limitations exist, our findings provide a preliminary framework for future investigations into this topic. By expanding the range of network structures used in future studies and focusing on specific layers and modules within neural networks, we advanced our understanding of visual illusions. This work provided insights for improving neural network design. We hope that our research serves as a valuable foundation for other researchers in the field of visual illusions.
